# Nanorepair medicine for treatment of organ injury

**DOI:** 10.1093/nsr/nwae280

**Published:** 2024-08-10

**Authors:** Han Wang, Jessica C Hsu, Wenyu Song, Xiaoli Lan, Weibo Cai, Dalong Ni

**Affiliations:** Department of Orthopaedics, Shanghai Key Laboratory for Prevention and Treatment of Bone and Joint Diseases, Shanghai Institute of Traumatology and Orthopaedics, Ruijin Hospital, Shanghai Jiao Tong University School of Medicine, Shanghai 200025, China; Departments of Radiology and Medical Physics, University of Wisconsin-Madison, Madison, WI 53705, USA; Departments of Radiology and Medical Physics, University of Wisconsin-Madison, Madison, WI 53705, USA; Department of Nuclear Medicine, Union Hospital, Tongji Medical College, Huazhong University of Science and Technology, Wuhan 430073, China; Hubei Province Key Laboratory of Molecular Imaging, Wuhan 430022, China; Key Laboratory of Biological Targeted Therapy of the Ministry of Education, Wuhan 430073, China; Department of Nuclear Medicine, Union Hospital, Tongji Medical College, Huazhong University of Science and Technology, Wuhan 430073, China; Hubei Province Key Laboratory of Molecular Imaging, Wuhan 430022, China; Key Laboratory of Biological Targeted Therapy of the Ministry of Education, Wuhan 430073, China; Departments of Radiology and Medical Physics, University of Wisconsin-Madison, Madison, WI 53705, USA; Department of Orthopaedics, Shanghai Key Laboratory for Prevention and Treatment of Bone and Joint Diseases, Shanghai Institute of Traumatology and Orthopaedics, Ruijin Hospital, Shanghai Jiao Tong University School of Medicine, Shanghai 200025, China

**Keywords:** nanomedicine, organ injury, reactive oxygen species, inflammation, drug administration

## Abstract

Organ injuries, such as acute kidney injury, ischemic stroke, and spinal cord injury, often result in complications that can be life-threatening or even fatal. Recently, many nanomaterials have emerged as promising agents for repairing various organ injuries. In this review, we present the important developments in the field of nanomaterial-based repair medicine, herein referred to as ‘nanorepair medicine’. We first introduce the disease characteristics associated with different types of organ injuries and highlight key examples of relevant nanorepair medicine. We then provide a summary of existing strategies in nanorepair medicine, including organ-targeting methodologies and potential countermeasures against exogenous and endogenous pathologic risk factors. Finally, we offer our perspectives on current challenges and future expectations for the advancement of nanomedicine designed for organ injury repair.

## Teaser

This paper defines and reviews the emerging cross-disciplinary field of nanorepair medicine which intends to explore the applicability of nanomaterials for the repair of organ injuries.

## INTRODUCTION

Senescence and various stimuli, such as trauma, radiation, toxins, and viral infections, can inflict severe damage upon vital organs, resulting in a decreased quality of life or even death [1,2]. Organ injuries can affect people of all ages and health conditions; thus, treatments for organ injures have become an important scientific pursuit aimed at improving population health. Intriguingly, certain species, including salamander and planaria, have demonstrated remarkable regenerative capacities pertaining to organ repair [3,4]. For example, salamanders can regenerate their legs, tails, spinal cords, and even parts of their brains. In stark contrast, higher mammals, including humans, have displayed markedly limited self-regenerative capabilities [5]. Consequently, when humans suffer organ injuries, the prevailing clinical approach revolves around the options of either repairing the existing damaged organs or replacing them with donor organs [6]. Given the scarcity of organ donors and the considerable medical costs associated with transplantation procedures, the strategy of repairing damaged organs emerges as the more pragmatic and sustainable choice.

A multitude of organ injuries exist, including acute kidney injury (AKI), ischemic stroke, and inflammatory bowel disease (IBD), among others. These injuries often exhibit similar disease characteristics, particularly an activated inflammatory microenvironment and an excess of reactive oxygen species (ROS) [7–9]. Generally, when exposed to various pathologic risk factors, damaged cells or pathogens trigger the recruitment of inflammatory cells, which in turn secrete various cytokines and ROS to attract more inflammatory cells [9–11]. While controlled inflammation can facilitate organ injury repair, uncontrollable inflammation can lead to an overproduction of ROS, exacerbating organ damage [11]. Timely ROS scavenging and anti-inflammatory interventions are crucial to disrupt this detrimental cycle and promote organ injury repair. Despite these commonalities in pathogenesis, each organ injury type is also characterized by its own unique underlying pathological processes that are intricate and not well-understood. These complexities pose significant difficulties in clinical treatment applications. Many drugs exhibit suboptimal therapeutic efficacy (e.g. 5-aminosalicylic acid for IBD), as well as issues such as poor bioavailability/pharmacokinetics and/or undesired organ distribution [7,12,13]. Furthermore, specific and effective medications remain absent for certain organ injuries, such as AKI and Alzheimer's disease, thereby leaving only symptomatic and supportive treatments to alleviate symptoms [14]. Hence, there is a pressing need to develop innovative strategies for treating organ injuries.

Certainly, repair strategies should consider both the similarities and unique characteristics of each organ injury. The structure and function of nanomaterials can be finely tuned to exhibit different behaviours that meet the clinical needs with satisfactory results. Over the past decade, many studies have recognized common underlying disease mechanisms among various organ injuries (e.g. overproduction of ROS). Nanomaterials like metal-organic frameworks (MOFs), ceria nanoparticles (NPs), and polyoxometalate clusters, have been synthesized and applied in the repair of organ injuries through the scavenging of ROS [15–18]. In addition, a subset of these studies has presented suitable methods and strategies tailored to the specific attributes of individual organ injury type (e.g. organ targeting). These nanomaterials have demonstrated superior therapeutic efficacy compared to conventional pharmaceutics and clinical treatments due to their enhanced pharmacological properties and/or improved targeting capabilities. The use of nanomaterials to ameliorate organ injuries has given rise to a growing field known as nanomaterial-based repair medicine, which we refer to as ‘nanorepair medicine’ throughout this review.

**Figure 1.**
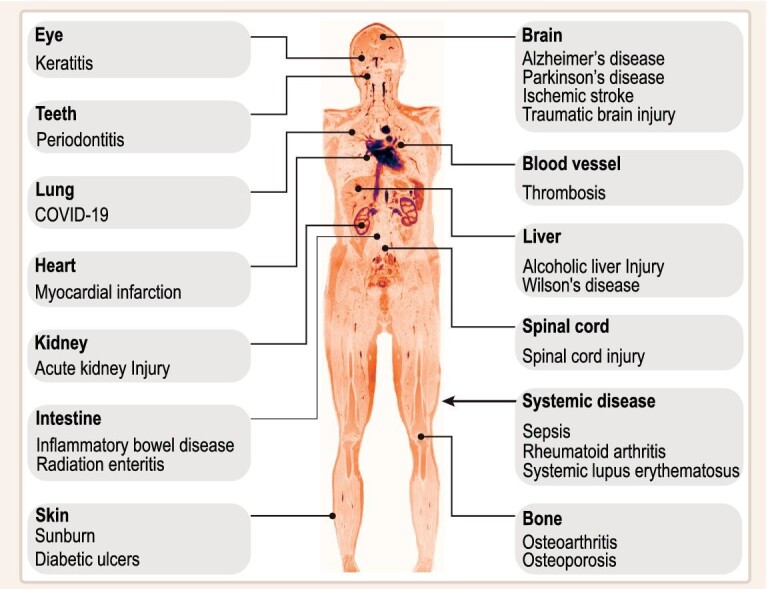
Representative applications of nanomaterials used in organ injuries. This pseudo-colour magnetic resonance image was obtained from a young Asian woman with permission.

In this review, we aim to introduce the concept of nanorepair medicine—an emerging cross-disciplinary field that intends to explore the applicability of nanomaterials for the repair of organ injuries. To provide a comprehensive understanding of nanorepair medicine, we first present a summary of the relevant applications of nanomaterials in organ injury repair, with a focus on addressing intractable and/or common diseases. The 22 specific organ injuries related to our discussion are illustrated in Fig. 1. From these applications, we then outline valuable targeting and treatment strategies. Last, we offer our perspectives on the current challenges and future directions within the field of nanorepair medicine. The overarching goal of this review is to inspire new ideas for organ injury treatments and foster collaboration and knowledge exchange between clinicians and material scientists.

## APPLICATIONS OF NANOREPAIR MEDICINE

### Cardiovascular diseases

Cardiovascular diseases (CVDs), represented by myocardial infarction (MI) and ischemic stroke, account for more than 17 million deaths worldwide each year [19]. Generally, high-risk factors such as smoking, alcohol consumption, stress, obesity, hypertension, and diabetes promote the formation of atherosclerotic plaques, a process that can span decades [19,20]. Atherosclerotic plaques can become dislodged due to stimuli like emotional fluctuations and temperature variations, potentially leading to the blockage of heart and cerebral vessels and the subsequent onset of MI and ischemic stroke [21,22]. While intravascular stent implantation is recommended for the treatment of infarctions, serious complications such as heart failure and hemiplegia still often occur.

Recently, a biodegradable polymer, loaded with polyvinylpyrrolidone (PVP)/H_2_O_2_ adduct and modified with catalase (CAT) and platelet membrane on the surface, was developed to treat MI. This nanomaterial was designed to target MI and slowly degrade to release CAT, which in turn reverses oxygen deficiency by converting H_2_O_2_ to O_2_ [23]. This approach is aimed at reducing cell death by delivering essential nutrients. In addition, during ischemia-reperfusion, blood reperfusion can lead to an overproduction of ROS, resulting in cellular damage. In a recent study, ceria NPs loaded with dl-3-n-butylphthalide (NBP) were developed to treat ischemic stroke in middle cerebral artery occlusion/reperfusion (MCAO/R) murine models. Ceria serves as a scavenger for ROS, while NBP improves neurovascular functions by promoting angiogenesis [24]. Similarly, another study used human serum albumin (HSA) modified Mn_3_O_4_ NPs to treat MCAO/R mice by scavenging ROS [25].

In the prevention of infarction, the removal of thrombosis is a common approach. Thrombolytic agents, such as plasminogen activator (PA) drugs, have been widely used to dissolve thrombi. To enhance target delivery and reduce off-target effects of thrombolytic agents, some novel drug carrier systems have been developed. For example, poly(lactic-co-glycolic acid) (PLGA) microaggregates loaded with tissue PA (tPA) were designed to treat thrombosis. The abnormally high fluid shear stress within the area of thrombus could break down the microaggregates and expose tPA [26]. Another study engineered PLGA NPs camouflaged with platelet membranes to deliver recombinant tissue PA (rt-PA) to the site of thrombosis [27].

### Digestive and excretory system injury

Digestive and excretory system injury usually happens in clinics as this system is used for nutrition uptake or excretion of metabolic waste. For instance, liver will be damaged upon a variety of factors such as viruses, alcohol consumption, metal ions, and certain drugs [28,29]. Prolonged liver injury can lead to fibrosis or cirrhosis of the liver, ultimately culminating in liver failure [29–31]. Hence, timely detoxification is crucial in preventing liver damage. Recently, Fe-based MOFs (MIL-101) loaded with alcohol oxidase and aldehyde dehydrogenase were utilized in treating alcoholic liver injury. These loaded enzymes effectively convert alcohol to acetic acid, thereby avoiding the harmful effects of aldehyde [32]. In the case of Wilson's disease, a rare condition characterized by abnormal accumulation of copper in the liver and other vital organs, Au@ZnMoS_4_ NPs were developed to selectively remove copper ions from HepG2 cells (or hepatocellular carcinoma) in the presence of other endogenous and metabolically essential metal ions [33].

Enteritis, which can be induced by microbes, radiation, toxins, autoimmune disorders, and even, more recently reported, microplastics, is a prevalent disease with a high global morbidity rate [34,35]. Within the spectrum of enteritis, IBD represents a subtype characterized by chronic inflammation that predominantly affects certain segments of the gastrointestinal tract, often giving rise to extraintestinal complications [7]. While clinical treatments for IBD encompass a range of immunosuppressants, amino salicylates, and steroids, the prolonged use of these drugs may result in a series of side effects, including autoimmune responses and susceptibility to viral infections [36]. Although the pathogenesis of IBD is poorly understood, it is well established that an up-regulation of ROS levels occurs within IBD-affected tissues. Current nanorepair medicine mainly focuses on scavenging ROS for treating IBD. A study demonstrated the potential of manganese-based MOFs (PCN-222-Mn) loaded with Pt NPs to mimic the activity of CAT and superoxide dismutase (SOD), effectively scavenging ROS in mice afflicted with dextran sulfate sodium (DSS) and 2,4,6-trinitro benzene sulfonic acid induced IBD [37]. More recently, our group developed an oral formulation of low-valence molybdenum nanodots to scavenge harmful ROS in DSS induced IBD mice [36]. Furthermore, the modulation of gut microbiota presents a prospective avenue for IBD intervention [7]. For example, it was reported that WO_3_ NPs can treat IBD by inhibiting Enterobacteriaceae growth [38]. Radiation enteritis, another prevalent form of intestinal injury, often occurs after radiation therapy to the abdominal and pelvic regions [39]. ROS generated by ionizing radiation impairs the DNA and membrane system of intestinal cells, leading to subsequent cell death [39]. Amifostine is a clinically-approved, radioprotective prodrug that scavenges ROS when converted into an active sulfhydryl compound by phosphatase. However, the oral administration of amifostine is rendered impractical as it deteriorates and becomes inactive upon exposure to gastric acid. Recently, *Spirulina Platensis* was used as a natural delivery carrier for amifostine, enabling the development of an oral formulation that supports gut microbiota homeostasis in mice treated with 12 Gy X-ray radiation [12].

Acute kidney injury (AKI), a common occurrence especially among hospitalized patients, denotes a sudden and often reversible reduction in kidney function, as evidenced by increased creatinine levels or decreased urine volume [14]. The current management of AKI primarily involves symptomatic and supportive treatments, with severe cases necessitating renal replacement therapy [14]. The excessive production of ROS contributes to the initiation and progress of AKI [40]. Thus, alleviating oxidative stress has become a promising repair strategy for AKI. Ultrasmall (sub-5 nm) antioxidant NPs are usually considered and used for AKI treatment due to their propensity to target the kidneys [41]. For instance, our group developed ultrasmall molybdenum-based polyoxometalate (POM) nanoclusters to treat mice with glycerol-induced AKI [18]. Molybdenum atoms in POM can readily scavenge ROS by shifting between Mo^5+^ and Mo^6+^ valence states during the redox process. Similarly, ultrasmall ceria NPs, selenium-doped carbon quantum dots (SeCQDs), and tungsten-tannic acid nanocomplexes have also demonstrated efficacy in AKI treatment by eliminating a broad array of harmful ROS [42–44]. Interestingly, some large-sized nanomaterials with specific shapes can target the kidneys. For instance, rectangular DNA origami nanostructures (DONs) (∼90 nm) served as an effective repair agent for AKI via preferential renal accumulation and potent ROS scavenging capability [40]. Notably, delivering essential redox cofactors has shown promise in AKI treatment. Recently, ultrasmall Fe_3_O_4_ NPs modified with nicotinamide mononucleotide (NMN), a precursor of NAD^+^, were used to treat AKI, while enabling magnetic resonance imaging (MRI) of the kidneys [45].

### Skin injury

As the body's first line of defence and outermost barrier, skin plays a significant role in multiple facets of human health. Many internal factors (e.g. diabetes and immunity) and external factors (e.g. radiation, pressure, and pathogenic organisms) are known to induce complex skin injuries [46]. The preferred therapeutic strategy is to prevent skin damage from these factors. In daily life, inorganic micronized particles (e.g. ZnO and TiO_2_) and organic sunscreen (e.g. padimate O) have been widely used to avoid sunburn and mitigate the risk of skin injuries by absorbing or reflecting ultraviolet (UV) radiation. However, organic sunblock products may raise safety concerns due to potential cutaneous absorption. To overcome this issue, polylactic acid-hyperbranched polyglycerol (PLA-HPG)-based NPs loaded with padimate O were recently developed as a sunblock. This formulation was designed to prevent intra-epidermal or follicular penetration upon topical application [47]. When dealing with existing skin injuries, the management of chronic wounds can be particularly challenging. Diabetic ulcers, a prevalent form of chronic wounds, often result from chronic foot trauma and may even progress to the point of lower limb amputation [48]. Factors such as oxidative stress, high glucose levels, and concomitant infections within the wound microenvironment can significantly impede the natural repair process. Recently, copper-based MOFs (MOF-818) were developed to mimic the activities of SOD and CAT for the treatment of diabetic ulcers [15]. Additionally, oxygen-deficient molybdenum-based nanodots (MoO_3-x_) demonstrated the ability to scavenge ROS while simultaneously promoting cell regeneration in diabetic ulcers [49].

### Orthopedic diseases

Osteoarthritis (OA) is the most commonly occurring joint disorder. Inflammation in OA is associated with the activation of macrophages, which triggers an overproduction of ROS, subsequently leading to chondrocyte apoptosis [50]. Traditionally, OA treatment primarily focuses on pain management, with joint replacement reserved for end-stage disease. Current nanomaterial development mainly focuses on stem cell differentiation, clearance of senescent cells, and reversal of macrophage polarization. For example, PLGA loaded with nanosized magnesium oxide (MgO) has been developed for OA treatment. The MgO could release Mg^2+^ to promote chondrogenic differentiation of bone marrow mesenchymal stem cells (BMSCs) [51]. Another research applied aptamer-functionalized liposome loaded with senolytic (i.e. dasatinib and quercetin) to treat OA through eliminating senescent fibroblast-like synoviocytes [52]. More recently, Fe_2_O_3_ NPs modified transient receptor potential vanilloid family member 1 (TRPV1) monoclonal antibody were developed for OA treatment, which could target TRPV1 overexpressed macrophages. Upon alternating magnetic field, these Fe_2_O_3_ NPs could produce heat to activate TRPV1 and then reverse the macrophage inflammatory polarization [53].

Osteoporosis is a systemic skeletal disease characterized by low bone mass and microarchitectural deterioration of bone tissues. In the process of osteoporosis, hyperactive osteoclasts secrete acids and enzymes to dissolve calcium and collagen, respectively [54]. Clinically, diphosphonate is used to treat osteoporosis by inhibiting the activity of osteoclasts. However, it may induce serious side effects, including nephrotoxicity, osteonecrosis, and osteosarcoma genesis, among others. Current nanorepair medicine mainly focuses on stem cell differentiation for reversing osteoporosis. In a recent study, it was found that nitric oxide (NO) could potentiate bone-forming osteoblasts in ovariectomized (OVX) rats with osteoporosis. To improve the stability and release kinetics of a NO donor (NONOate), capric acid and octadecane were used to encapsulate NONOate. The as-prepared micelles showed long-lasting activity with the potential to reverse osteoporosis [55]. In another example, the Fe_2_O_3_ NPs modified with alendronate could target the bone and scavenge ROS to promote osteogenic differentiation in the model of OVX rats with osteoporosis [56].

### Other organ injuries

Pathogenic microorganisms, such as bacteria, fungi, and viruses, can induce infections and cause damage to multiple organs. Thus, eliminating these microbes is crucial for treating infectious injuries. Antibiotics and antifungal agents have traditionally been used for infection treatment, but the emergence of drug-resistant microorganisms poses a major challenge [57,58]. Currently, nanomaterials with the ability to generate ROS have been developed to combat bacteria and fungi, potentially avoiding issues related to drug resistance. For example, ethylenediaminetetraacetic acid (EDTA) modified Ag-Cu_2_O NPs were used to treat *Candida albicans* induced fungal keratitis. EDTA was effective in disrupting fungal cell walls and inhibiting filamentation and biofilm formation, while Ag-Cu_2_O generated ROS and silver ions to further eradicate *C. albicans* [59]. In another example, Fe_2_O_3_-modified porphyrinic Cu-MOFs were designed to treat periodontitis. The heterostructure of the nanomaterial, along with the release of Cu^2+^ and Fe^3+^, increased the effect of photodynamic therapy against various oral pathogens [60]. Until now, the primary focus of research endeavors in the realm of viral infection treatments has been directed toward neutralizing viruses. For example, coronavirus disease 2019 (COVID-19) is a highly contagious viral illness caused by severe acute respiratory syndrome coronavirus 2 (SARS-CoV-2) [61,62]. SARS-CoV-2 is known to enter lung cells via the angiotensin-converting enzyme 2 (ACE2) receptor [63]. Inhalable ACE2 nanodecoys synthesized from the extrusion of human lung spheroid cells were shown to neutralize SARS-CoV-2 and protect lung cells from viral infections in mice and cynomolgus macaques [64]. In another study, ultrathin CuInP_2_S_6_ nanosheets with high binding affinity toward wild-type SARS-CoV-2 and its variants (Delta and Omicron) demonstrated protection against viral infections in human ACE2-transgenic mice [65]. Additionally, supplying essential redox cofactors has shown promise in the treatment of infectious injuries. In a recent study, lipid coated calcium phosphate (CaP) NPs loaded with NAD^+^ and lipid coated ZIF-8 NPs loaded with a reduced form of NAD^+^ (NADH) were developed to treat sepsis, an infection often accompanied by a systemic inflammatory response syndrome, by improving cellular energy supply, suppressing inflammation, and preventing cell death [66].

Apart from external microorganisms, external trauma can also induce serious organ injuries, especially nerve injuries such as spinal cord injury (SCI) and traumatic brain injury (TBI). Nanomaterials have shown promise in treating these injuries by delivering a neuromodulator and scavenging ROS. For example, squalenoyl adenosine NPs were developed to address the short plasma half-life of adenosine and its limited ability to cross the blood-spinal cord barrier, thus making them a promising SCI repair agent [13]. In another study, Mn doped Ag_2_Te QDs were used to treat TBI. Manganese served as both CAT and SOD to effectively scavenge ROS, while Ag_2_Te provided near-infrared (NIR) image-guided therapy [67].

Neurodegenerative diseases, such as Alzheimer's disease (AD) and Parkinson's disease (PD), often stem from nerve injuries that result in irreversible memory loss and a progressive decline in cognitive functions [68]. The pathogenesis of PD and AD is complex and not fully understood. Current evidence suggests that the polymerization of amyloid β-peptides (Aβ) induces the generation of excess ROS, leading to neuronal death in AD [68]. A recent study found that nickel or cobalt doped POM could cross the blood-brain barrier (BBB) and inhibit Aβ fibril formation by binding to the histidine site of Aβ [69]. Another study reported the use of triphenylphosphine (TPP) conjugated ceria NPs to target and scavenge mitochondrial ROS in 5XFAD transgenic mice [70]. In the case of PD, the regulation of pyroptosis has emerged as a potential therapeutic method. Recently, Prussian blue NPs served as a pyroptosis inhibitor for treating PD via ROS scavenging and inhibition of microglial NLRP3 inflammasome [71].

Autoimmune diseases, such as rheumatoid arthritis (RA) and systemic lupus erythematosus (SLE), are caused by a hyperactive immune system attacking and injuring normal organs [22,72,73]. Thus, suppressing the immune system is necessary for treating these diseases, and current nanorepair medicine mainly focuses on delivering immunosuppressants. For example, a recent study reported the use of PLGA NPs loaded with rapamycin and a ligand for the B cell inhibitory co-receptor CD22 (CD22L). These nanoparticles were designed to induce immune tolerance in regulatory T cells and B cells to a self-antigen for the suppression of RA [74]. In another study, polymeric NPs loaded with miR-125a were shown to restore the balance between effector and regulatory T cells (T_regs_) for the treatment of SLE [75]. A different approach used polymeric NPs loaded with an immunosuppressant (i.e. cyclosporine A) and gambogic acid to treat SLE. Gambogic acid could target gut-associated lymphatic tissue and enhance the delivery of cyclosporine A to the systemic lymphatic circulation [73].

## CURRENT STRATEGIES IN NANOREPAIR MEDICINE

### Targeting certain organ injuries

**Figure 2.**
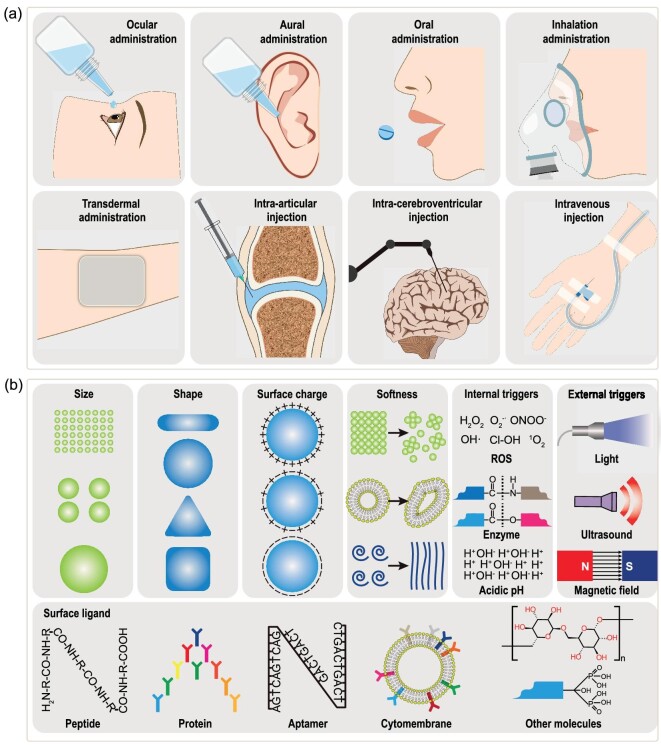
Targeting of organ injuries. (a) Various nanomaterial administration methods for targeting certain organs. (b) Physicochemical properties of nanomaterials that influence organ targeting.

There are two main factors that govern the ability of NPs to target particular organs. One is the administration route, and the other is the physicochemical properties of NPs. The typical administration methods of NPs include intra-cerebroventricular injection, intra-articular injection, intravenous injection, transdermal administration, ocular administration, aural administration, oral administration, and inhalation administration, among several others (Fig. 2a) [76]. Local administration allows NPs to concentrate in certain organs or injured tissues. For example, transdermal administration is suitable for the direct treatment of skin injuries, while eye drops are preferred for ocular injuries. Selecting the appropriate administration route is necessary to facilitate the transport of nanomaterials across biological barriers. For instance, due to the presence of the BBB, nanomaterials rarely reach the brain through intravenous injection [77,78]. On the other hand, lumbar puncture or intra-cerebroventricular injection allows for direct delivery to the brain [71].

The physicochemical properties of NPs, such as size, shape, surface charge, softness, surface ligand, and stimuli-responsiveness, strongly influence their ability to target specific organs (Fig. 2b). Generally, upon intravenous injection, NPs with a large size (>20 nm) are nonspecifically taken up by the mononuclear phagocyte system. This can be particularly beneficial when solely targeting the liver or spleen. On the other hand, ultrasmall NPs (<5 nm) can easily pass through the renal glomeruli and be rapidly cleared through the urinary system, which can be beneficial for kidney targeting [41]. NP shapes also affect their organ distribution profiles. For instance, rectangular DONs, despite having a size (∼90 nm) larger than the renal filtration threshold, can accumulate in the kidneys due to their unique morphology [40]. Furthermore, neutrally and negatively charged surfaces tend to deter the adsorption of serum proteins, thus increasing the stability and blood circulation half-life of NPs [41]. On the contrary, NPs with a positively charged surface can target adipose tissues due to their cationic nature [79]. Soft NPs, such as micelles, liposomes, and polymers, have a less rigid structure that enables them to contort when subjected to shear forces (e.g. blood flow or tissue interstitial pressure). This property allows them to be used for targeting thrombi or enhancing tissue penetration [80].

Surface ligands, including peptides, proteins, aptamers, cytomembranes, and other molecules (Fig. 2b), have been widely utilized in organ-targeting applications [33,73,74,81,82]. Specific sequence peptides can exhibit unique targeting abilities. For example, an ischemic heart targeted peptide can assist NPs in targeting CVDs [83]. Fibrin-targeting peptides can guide NPs to thrombus sites [84]. Peptide sequences derived from sodium channels can increase the targeting of local anesthetics to site-1 sodium channel blockers [85]. Proteins like antibodies and ligandin also have the ability for organ targeting. For instance, anti-CD4-antibody modified NPs can target CD4^+^ cells [86]. In addition, Fas-ligand modified NPs can target activated T cells with overexpressed Fas (a cell death receptor) [87]. Aptamers are composed of oligonucleotide sequences that can target or recognize ions, viruses, cells, and tissues with high specificity [88,89]. For example, a chemokine C-C motif ligand 21 specific aptamer with lymph node targeting capability was developed [90]. Furthermore, surface modification with cytomembranes obtained from erythrocytes, mesenchymal stromal cells, and neutrophils has recently been demonstrated. This coating strategy allows for effective targeting of certain organs due to the presence of various surface receptors, while ensuring safety and immune compatibility [16,91,92]. There are also other molecules that can be used as organ-targeting ligands. For example, diphosphonate-modified NPs can target bone tissues [93,94]. Carbohydrate molecules like CD22L (a glycan ligand) can target B cells, while glucose-conjugated NPs can bypass the BBB by targeting glucose transporter-1 [74,95].

The responsiveness of nanomaterials to various stimuli plays an important role in achieving precise organ targeting. Nanomaterials can respond to both internal stimuli (e.g. ROS, enzymes, and acidic pH) and external stimuli (e.g. light, ultrasound, and magnetic field) [20]. For example, inflammatory microenvironments typically constitute an abundance of ROS (mainly H_2_O_2_ and superoxide anion radicals) and metallomatrix proteinases (MMPs) [9,96]. Hence, NPs designed to be responsive to ROS and MMP can effectively target inflamed areas [96,97]. Moreover, certain organs may exhibit elevated levels of specific enzymes. For instance, the overexpression of esterase in the digestive juice can be exploited for precise targeting of the gastrointestinal tract [98]. In addition, pH-responsive NPs are particularly well-suited for targeting acidic conditions presented in some injuries (e.g. osteoporosis) and cellular components (e.g. endosomes and lysosomes) [81,99]. As for external triggers, light is often utilized in applications such as photodynamic therapy, photothermal conversion, and photolysis [59,60,83,100]. Ultrasounds operate mainly through sonoluminescence or sonodynamic effects [101,102]. Last, magnetic fields can be employed to control the movement and location of magnetic NPs, enabling precise and direct targeting of specific organs [103].

### Targeting exogenous pathologic risk factors

**Figure 3.**
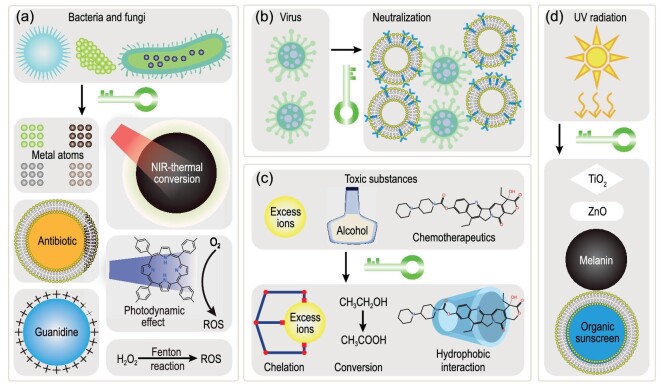
Targeting exogenous pathologic risk factors. There are many reported strategies to combat and eliminate (a) fungal and bacterial infections, (b) viruses, (c) toxins, and (d) damages from UV radiation.

Exogenous pathologic risk factors, such as fungi, bacteria, viruses, toxic substances, and radiation, can induce various organ injuries. Hence, strategies aimed at combating microbes, removing toxins, and preventing radiation damage are pivotal for addressing these factors. Treatment strategies for fungal and bacterial infections involve the use of antibacterial agents, heat, and harmful ROS (Fig. 3a). Using NPs to deliver antibacterial agents, such as metal ions (e.g. Bi, Ag, Mo and Ga), guanidine-based compounds, and antibiotics, is a common method to eradicate pathogens [49,104–107]. Certain NPs such as gold NPs and transition metal chalcogenides (e.g. MnSe_2_) can effectively elevate temperature through photothermal conversion upon NIR irradiation to treat infections [107,108]. ROS can also mediate bacterial killing via photodynamic effects (e.g. porphyrin-based NPs) or Fenton reactions (e.g. NPs containing Fe or Cu) [59,60,109]. Moreover, neutralizing viruses through interactions with NPs has been extensively studied as an antiviral approach, though further optimization is required (Fig. 3b) [64,65].

Toxic substances, such as heavy metal ions (e.g. Pb, Hg, Cd, Cu), excessive alcohol, organophosphorus compounds, and chemotherapeutics, are known to cause severe damages to nerve tissues, kidneys, and the liver [110,111]. Removing these toxins is necessary for treating related injuries, and recent research efforts have made significant progress in this regard (Fig. 3c). For instance, Au@ZnMoS_4_ NPs were developed to remove excess copper ions [33]. MIL-101 loaded with alcohol oxidase and aldehyde dehydrogenase were used to convert alcohol to acetic acid [32]. Cyclodextrin-based NPs were used to remove chemotherapeutics through supramolecular recognition [112].

Preventing radiation-induced damage represents a paramount treatment strategy for addressing organ injuries, yet research in this area has been somewhat limited, with a primary focus on reducing exposure to UV radiation. Commercial products such as TiO_2_ and ZnO powders have long been utilized for protection from UV radiation. Several nanomaterial formulations, such as melanin NPs (e.g. PDA and allomelanin) and NPs containing organic sunscreen, have been shown to shield the skin from the harmful effects of UV radiation by absorbing UV photons (Fig. 3d) [47,113].

### Targeting endogenous pathologic risk factors

**Figure 4.**
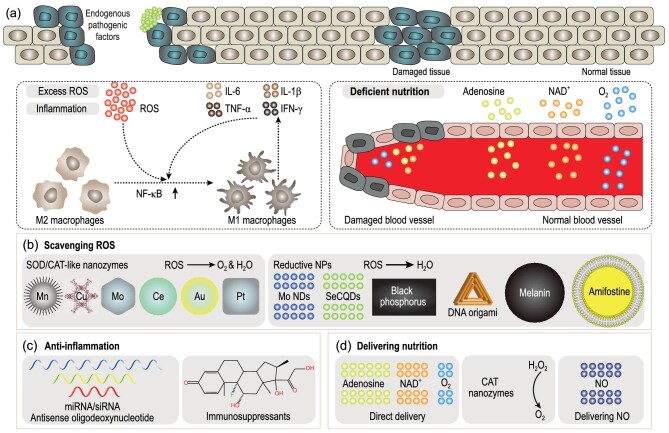
Targeting endogenous pathologic risk factors. (a) Several endogenous risk factors can exacerbate organ injuries and hinder the repair processes. Strategies are employed to (b) scavenge ROS, (c) induce anti-inflammatory effects, and (d) deliver vital nutrients to aid in organ injury repair.

Many endogenous pathologic risk factors including excess ROS, over-activated immunity, and deficient nutrition may impede the organ injury repair processes (Fig. 4a). The initiation and underlying pathological mechanism of inflammation can be quite complex. Various stimuli can lead to an overproduction of ROS. Excessive ROS can activate the nuclear factor κB (NF-κB) pathway and convert anti-inflammatory M2 macrophages into pro-inflammatory M1 macrophages. M1 macrophages are known to produce pro-inflammatory cytokines such as IL-6, IL-1β, interferon-γ (IFN-γ) and tumor necrosis factor α (TNF-α) [36]. These M1 macrophages can be further stimulated by cytokines, resulting in increased ROS production and an over-activated immune response that can damage surrounding normal cells [11,17,81]. Blood vessels can also be damaged during the inflammatory processes, thus hampering the delivery of essential nutrients to the injured organs. Hence, scavenging ROS, reducing inflammation, and delivering nutrients are the main strategies for addressing endogenous pathologic risk factors.

Scavenging excess ROS is a frequently used strategy in the field of nanorepair medicine. NPs with antioxidant properties can be classified into two main categories: SOD/CAT-like nanozymes and reductive NPs (Fig. 4b). SOD/CAT-like NPs mainly include manganese-based NPs (Mn-MOF, MnO_2_, Mn_3_O_4_) [16,25,114], copper-based NPs (MOF-818, Cu-tannic acid complex) [15,115], molybdenum-based NPs (POM, MoO_3-x_, MoS_2_) [18,49,116], cerium-based NPs (CeO_2_, Ce-MOF) [17,70,117], gold NPs [118], and platinum NPs [37], among others. Additionally, several physical variables including crystal facets, surface defects, and morphological structures (such as heterojunctions and nanopores) may influence the redox performance of nanozymes [119–121]. On the other hand, reductive NPs can scavenge ROS through reactions between ROS and low-valence atoms. As previously mentioned, SeCQDs [122], molybdenum nanodots (NDs) [36], black phosphorus [123], NPs loaded with amifostine [12], melanin NPs [113,124] and DNA origami [40] have been used to neutralize excess ROS.

Inflammation and oxidative stress from excessive ROS are interrelated, as each can promote the other in a toxic feedback loop. While direct ROS scavenging usually alleviates inflammation, there are alternative methods that can achieve the same goal. NPs loaded with certain small interfering RNA (siRNA) [125], miRNA [75], antisense oligodeoxynucleotide [126], and immunosuppressants (e.g. dexamethasone, betamethasone and cyclosporine A) [73,127] can mitigate an overactive immune system (Fig. 4c). These formulations have been used in the treatment of several autoimmune diseases.

Last, vital nutrient deficiencies can impede the repair of organ injuries. NPs loaded with NO donor can increase the supply of blood and nutrients to injured tissues [55,128]. Additionally, NPs loaded with essential nutrients required for proper cellular functions, such as NAD^+^, oxygen, and adenosine, can accelerate the regenerative and repair processes (Fig. 4d) [13,23,66].

## CONCLUSIONS AND FUTURE PERSPECTIVES

### Current state and challenges of nanorepair medicine

**Figure 5.**
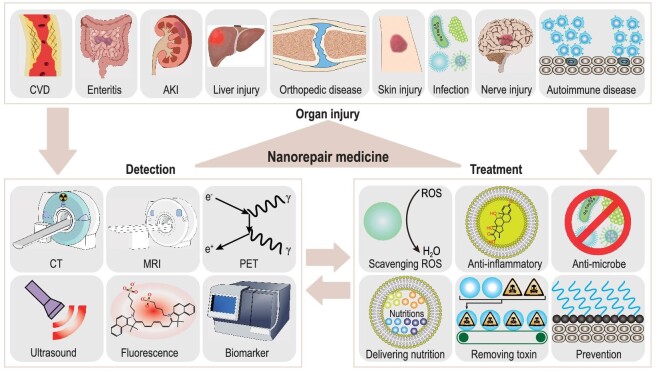
The outlook for nanorepair medicine.

In this review, we introduce the concept of nanorepair medicine. Recent progress in nanorepair medicine has been summarized in Table 1. The main treatment strategies of nanorepair medicine are also shown in Fig. 5. As an emerging field, interdisciplinary collaboration among clinicians, academic researchers, material scientists, radiologists, and industrial experts are crucial for the development and advancement of nanorepair medicine. For example, clinicians can share their experiences with current organ injury treatments and identify pitfalls and potential alternatives. Scientists in both academia and industries can then make improvements to the design of nanomaterials. Evidently, there is a growing research interest toward nanorepair medicine, and interdisciplinary collaboration has now become a prominent trend and a tangible reality. This evolving landscape holds the promise to attract additional resources to drive the field forward.

**Table 1.** Summary of several nanorepair medicine for the treatment of various organ injuries.

**Organ**	**Type of injury**	**Related nanomaterials**	**Treatment strategy**	**Reference**
Skin	Diabetic ulcer	MOF-818	Scavenging ROS	[15]
		MoO_3-x_	Scavenging ROS; Promoting cell regeneration	[49]
	Sunburn	PLA-HPG NPs loaded with Padimate O	Preventing UV	[47]
Intestine	Inflammatory bowel disease	Mo nanodots	Scavenging ROS	[36]
		Pt@PCN222-Mn	Scavenging ROS	[37]
		WO_3_	Inhibiting Enterobacteriaceae	[38]
	Radiation enteritis	*Spirulina platensis* loaded with amifostine	Scavenging ROS	[12]
Kidney	Acute kidney injury	POM	Scavenging ROS	[18]
		Ultrasmall ceria	Scavenging ROS	[42]
		SeCQDs	Scavenging ROS	[43]
		Tungsten-tannic acid nanocomplex	Scavenging ROS	[44]
		DNA origami nanostructures	Scavenging ROS	[40]
		Fe_3_O_4_ loaded with NMN	Delivering NMN	[45]
Lung	COVID-19	ACE2 nanodecoys	Neutralizing virus	[64]
		CuInP_2_S_6_	Neutralizing virus	[65]
Liver	Alcoholic liver injury	MIL-101 loaded with alcohol oxidase and aldehyde dehydrogenase	Converting alcohol to acetic acid	[32]
	Wilson's disease	Au@ZnMoS_4_	Removing copper ions	[33]
Bone	Osteoporosis	Capric acid and octadecane loaded with NONOate	Delivering NO	[55]
		Alendronate-modified Fe_2_O_3_ NPs	Scavenging ROS	[56]
	Osteoarthritis	PLGA loaded with MgO	Stem cell differentiation	[51]
		Liposome loaded with dasatinib and quercetin	Clearance of senescent cells	[52]
		TRPV1 antibody-modified Fe_2_O_3_ NPs	Reversal of macrophage polarization	[53]
Heart	Myocardial infarction	Polymer loaded with CAT and PVP/H_2_O_2_	Delivering O_2_	[23]
Blood vessel	Thrombosis	PLGA loaded with tPA	Thrombolysis	[26]
		Platelet membranes modified PLGA loaded with rt-PA	Thrombolysis	[27]
Brain	Alzheimer's disease	Ni or Co doped POM	Inhibiting Aβ fibril formation	[69]
		TPP conjugated ceria	Scavenging ROS	[70]
	Parkinson's disease	Prussian blue	Scavenging ROS; Inhibiting pyroptosis	[71]
	Ischemic stroke	Ceria loaded with NBP	Scavenging ROS; Promoting angiogenesis	[24]
		HSA-Mn_3_O_4_	Scavenging ROS	[25]
	Traumatic brain injury	Mn doped Ag_2_Te QDs	Scavenging ROS	[67]
Spinal cord	Spinal cord injury	Squalenoyl adenosine	Delivering adenosine	[13]
Eye	Keratitis	EDTA modified Ag-Cu_2_O	Anti-fungal	[59]
Teeth	Periodontitis	Fe_2_O_3_-modified Cu-MOFs	Anti-bacteria	[60]
Systemic disease	Sepsis	Lipid coated CaP or ZIF-8 loaded with NAD(H)	Delivering NAD(H)	[66]
	Rheumatoid arthritis	PLGA loaded with rapamycin and CD22L	Immunosuppression	[74]
	Systemic lupus erythematosus	Polymers loaded with miR-125a	Immunosuppression	[75]
		Polymers loaded with gambogic acid and cyclosporine A	Immunosuppression	[73]

Despite the progress made, several challenges still need to be addressed. First, developing more diverse and effective treatments for various organ injuries is key to advancing the field. However, progress in this regard depends on current breakthroughs in biological and preclinical research, given that the pathogenesis for most organ injury types is not completely clear or well-understood. Second, extensive studies on biodistribution, long-term toxicity, metabolism, clearance, and related aspects are necessary before nanomedicine can be considered for clinical trials. A deeper understanding of the delivery and degradability of nanomaterials *in vivo* is also warranted. As research advances, we anticipate that these challenges and obstacles to clinical translation can be overcome.

### Outlook for nanorepair medicine

Nanorepair medicine has good economic prospects with many opportunities for commercial consideration because the versatility and flexibility of nanomaterial designs allow for various disease therapies. Owing to an aging population and increasing clinical demands, the role of nanomedicine in organ injury repair is anticipated to grow even larger. Thus, more investments and fundings are expected in the coming years to drive the advancements and commercialization of novel formulations.

In the future, nanorepair medicine is expected to complement and integrate with existing treatment procedures for organ injuries, offering a more effective approach to disease management. Organ injuries can be diagnosed using various imaging techniques, such as computed tomography (CT), MRI, and positron emission tomography (PET). These detection methods serve a dual purpose, not only aiding in diagnosis but also in monitoring the effectiveness and outcome of therapeutic interventions. Consequently, ensuring high-quality imaging is just as vital as the treatment itself. Indeed, injury detection is emerging as a prominent area of interest within the field of nanorepair medicine. This growing emphasis holds particular significance in the context of enabling future clinical translation, as advanced detection techniques and imaging probes can provide molecular insights into diseases and facilitate tracking of drug delivery as well as monitoring of treatment responses (Fig. 5). Currently, there exists a limited but notable body of groundbreaking research publications on topics such as radiolabelled NPs, nanoscale contrast agents, and biomarker detection [17,45,129]. Newly emerging treatment and detection strategies, when combined, can create a synergistic theranostic platform, opening new avenues for advancement and inspiring further innovations in the field of nanorepair medicine.
